# Technological limitations of solid-source chemical vapor deposition of van der Waals heterostructures

**DOI:** 10.1038/s41598-025-13921-4

**Published:** 2025-08-05

**Authors:** Jakub Sitek, Wojciech Sitek, Ben R. Conran, Xiaochen Wang, Clifford McAleese, Anna Kaleta, Slawomir Kret, Iwona Pasternak, Mariusz Zdrojek, Wlodek Strupinski

**Affiliations:** 1 Faculty of Physics, Warsaw University of Technology, Koszykowa 75, 00-662 Warsaw, Poland; 2 Center for Multidimensional Carbon Materials, Institute for Basic Science, UNIST-gil 50, Ulsan, 44919 South Korea; 3 Center for Terahertz Research and Applications CENTERA2, Warsaw University of Technology, Poleczki 19, 02-822 Warsaw, Poland; 4 Faculty of Mechanical Engineering, Silesian University of Technology, Konarskiego 18A, 44-100 Gliwice, Poland; 5 AIXTRON Ltd, Buckingway Business Park, Anderson Road, Swavesey, Cambridge, CB24 4FQ UK; 6 Institute of Physics, Polish Academy of Sciences, Al. Lotników 32/46, 02-668 Warsaw, Poland

**Keywords:** Chemical vapor deposition, 2D materials, Van der Waals heterostructures, CVD technology, Tungsten disulfide, Graphene, Graphene, Synthesis and processing, Two-dimensional materials, Characterization and analytical techniques, Design, synthesis and processing

## Abstract

The large-scale synthesis of van der Waals heterostructures (vdWHSs) is required to adopt these materials in electronic devices. However, the repeatable and controllable growth of vdWHSs has proven challenging. Here, we investigate the technological aspects of solid-source chemical vapor deposition (CVD) of two-dimensional heterostructures, with WS_2_/graphene and MoS_2_/graphene as examples. We show that by modification of one variable at least one another is unintentionally altered. For example, change in the growth pressure influences the evaporation rate of sulfur and shifts the position of one of the growth zones. We also perform a statistical screening of the 11 process parameters, indicating which of them impact the evaporation of the precursors. The screening indicates that the evaporation depends on weight of growth promoter (NaCl), growth temperature, precursors temperature, time difference between main and sulfur growth zones reaching the set temperatures, pressure, carrier gas flow, and process time. Finally, the five consecutive, identical growth processes show the seemingly inherent variability in synthesizing vdWHSs. We suggest that the high but limited airtightness of the CVD system or the substrate features can cause repeatability issues. Our study can facilitate future research on van der Waals heterostructures growth.

**Supplementary Information:**

The online version contains supplementary material available at 10.1038/s41598-025-13921-4.

## Introduction

Electronics based on two-dimensional (2D) materials, like semiconducting MoS_2_ or WSe_2_, are foreseen to contribute to CMOS + X (complementary metal-oxide-semiconductor with emerging technologies) in the near future and extend Moore’s law^1,2^. The electronic components can often be realized using a single 2D material^3^; however, usually different 2D materials stacked in van der Waals heterostructures (vdWHSs) are required to fully harness the potential of these materials^4,5^.

The early, groundbreaking works on the 2D-based electronics were based on the exfoliated flakes^3,6^. However, this approach limits the practical application of 2D materials, as exfoliation is not scalable and repeatable. Among methods that can be scaled and are highly reproducible, chemical vapor deposition (CVD) has proved to yield large-scale and high-quality layers^7^, including continuous monolayers^8^ and monocrystalline monolayers^9^, even though the initial experiments and the first commercially available samples were of low quality^10,11^.

Similarly, an intensive research effort is ongoing to synthesize single-crystal, wafer-scale heterostructures, but this goal is yet to be achieved^12,13^. A considerable number of works are still based on CVD utilizing solid precursors^14–16^, although modifications of CVD, like metal-organic chemical vapor deposition (MOCVD) started to be used more often in the research and are more viable industrially^17,18^. The problem of growth reproducibility and uniformity of 2D materials, both on a single wafer and between batches, has arisen in recent years^19^. Without repeatability and a high degree of control, it will be impossible to incorporate 2D materials in electronic devices, as electronics require very high yield^20^.

To date, there are no comprehensive studies of the controllability and reproducibility of van der Waals heterostructures. Here, we provide insight into the technology of CVD growth of van der Waals heterostructures using solid precursors. By synthesizing WS_2_/graphene and MoS_2_/graphene vdWHSs, we study the limitations of the solid-source CVD. We discuss that the process parameters are interdependent and a single variable cannot be changed without impacting other parameters, which hinders the controllability of the synthesis process. By performing a statistical screening of 11 growth parameters throughout 43 growth runs, we select the growth variables with a moderate and significant influence on the evaporation of precursors. We also show the apparent limits of reproducibility of solid-source CVD on the example of five, identical growth processes, and indicate the graphene substrate variability and air-tightness of the CVD system as the possible limiting factors.

## Experimental

### CVD system and growth process

The growth processes were conducted in a standard 55-mm inner (60-mm outer) diameter hot-wall reactor Carbolite Gero EZS-1200 with a quartz tube (1200 mm length) and an additional 100 or 220-mm homemade sulfur heater. The images of the complete setup are shown in Fig. S1. The system was pumped using an Agilent DS602 vacuum pump with an Edwards Ultragrade Performance 19 mineral oil. The vacuum pump was connected to the system by an 8-m-long vacuum tube to limit the oil back-diffusion. In the system, we used Viton O-rings. The quartz process tube protruded outside the end of the furnace thermal insulation by 210–260 mm in the upstream direction, depending on the used sulfur heater (100 and 220 mm, respectively). We used 75 × 15 × 10 mm quartz boats located 98 mm outside and 168 mm inside the furnace insulation for sulfur and tungsten/molybdenum oxide, respectively. The boats were placed inside an internal 400 × 25 mm quartz tube, which was positioned 205 mm from the furnace thermal insulation in the downstream direction.

The default growth process parameters were 900 °C, 950 mbar, 100 sccm Ar, and 15 min (counting from the main heating zone reaching the set temperature). Argon and nitrogen purity was 99.999%. Prior to the growth, we checked the furnace leak for 10 min at 50 mbar and then purged the furnace three times with 500 sccm Ar flow for three minutes (nine minutes total) with three minutes of pumping between purging steps (nine minutes total). The temperature ramping was set at approx. 35 °C/min. Typically, the furnace was cooled naturally from 900 to 700 °C, but afterward, the furnace cover was opened to let it cool rapidly. As precursors, we used sublimed sulfur (Chempur, p.a.), molybdenum oxide (Alfa Aesar, 99.95%), and tungsten oxide (TCI, 99.9%). NaCl (ACS, ACS reagent) was used as a growth promoter. In the default process, we used 1000 mg of sulfur and 50 mg of MoO_3_ or 200 mg of WO_3_, adding 10 mg of NaCl in the WS_2_ growths.

CVD graphene on sapphire was used as a substrate. Graphene was synthesized at 1560 °C for 4 min with methane as a carbon precursor on 2-inch c-plane sapphire wafers. The growth details can be found elsewhere^21^. The wafers were then cleaved into 5 × 5 mm pieces and placed on 500 × 20 mm quartz slab, used as a substrate support facilitating substrate loading and unloading.

### Characterization

We used a Bruker Dimension Icon atomic force microscope to determine the morphology of the samples. The topography of samples was measured in tapping mode using supersharp probes (tip radius ∼1 nm). SEM images were taken in Raith eLine plus electron-beam lithography SEM with in-lens and secondary electron detectors. The as-grown samples were characterized utilizing Raman spectroscopy and photoluminescence (PL). For this purpose, we used a Renishaw inVia Qontor Raman spectroscope in a backscattering configuration. Typically, measurements were done with a 532 nm laser, ×100 objective, 1800 lines/mm grating, and 2 mW laser power. Circularly polarized light was used to eliminate any symmetry-based phenomena. Raman microscope was also used to capture optical micrographs of the as-synthesized samples at ×50, ×200, and ×1000 magnification.

The WS_2_/graphene heterostructure interface was investigated with an FEI-Titan 80–300 transmission electron microscope operating at 300 kV, equipped with an image corrector. Before focused ion beam (FIB) preparation, the samples were covered with an amorphous carbon protective layer of approx. 5 nm. Subsequently, an FEI-Helios Nanolab 600 FIB was used to prepare the WS_2_/graphene/sapphire interface cross-section specimen by a SEM/FIB equipped with an OmniProbe nanomanipulator and platinum gas injection system (GIS). The standard polycrystalline platinum layer was deposited on the specimen to protect the material from damage during FIB lamella cutting out.

### Data analysis

The optical images of the heterostructures were processed using the ilastik software package, version 1.4.0rc8^22^. The software’s Random Forest algorithm was trained using four segments: monolayer, thin multilayer (approx. 2–8 layers), thick multilayer (more than approx. 8 layers), and substrate. Since batch processing could not always assign the features correctly, each image was manually adjusted and corrected using the PL data. Data was then exported and processed in ImageJ software, version 1.50i^23^. The minimum pixel size was set to 20 px.

Statistical analysis included the study of correlations between process parameters and the evaporation of sulfur and WO_3_. Correlations between all analyzed process variables were calculated to determine which have a significant effect on the process and to determine quasi-constant parameters. The calculations were performed using TIBCO Statistica v.13.3.

## Results and discussion

### Characteristics of the furnace

**Fig. 1:**
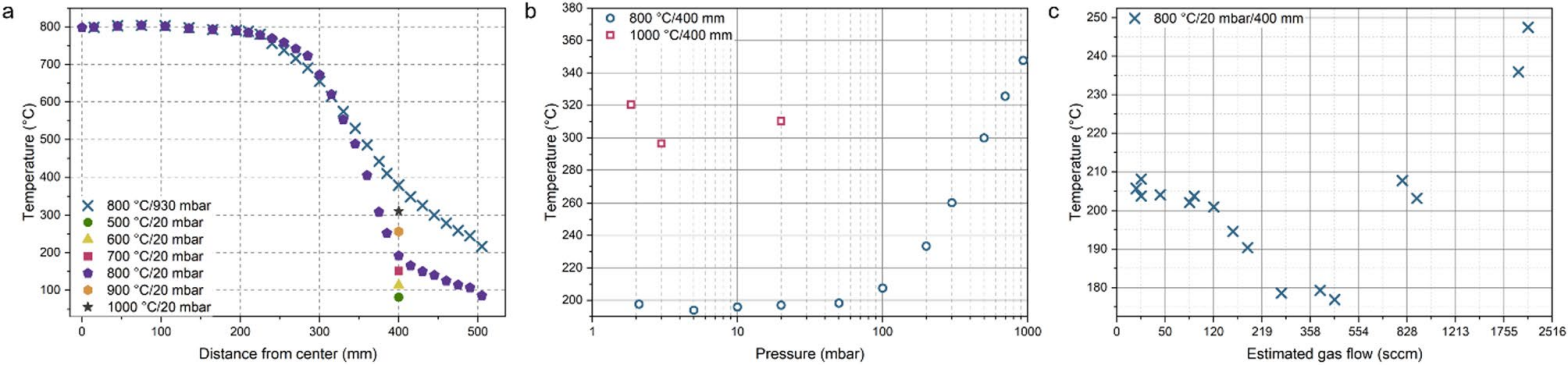
Characteristics of the CVD furnace: (**a**) impact of pressure and the main heating zone temperature on the temperature distribution; (**b**) a detailed impact of the pressure at a location 400 mm upstream from the heating zone center; (**c**) impact of the estimated gas flow at a location 400 mm upstream from the heating zone center.

The CVD system used in this study is manually controlled, i.e., gas, pressure, and temperature changes were not correlated and required input from the operator (Fig. S1). First, we measured the actual temperature inside the furnace, as the thermocouples controlling the heating coils are located in the furnace’s thermal insulation. We inserted the K-type thermocouple inside the quartz tube through a dedicated orifice. As presented in Fig. 1a, the actual temperature is close to the set values. However, there is a significant temperature gradient outside the heating zone. It is critical in the growth of 2D materials using solid precursors, as sulfur requires lower temperatures than the substrate and is intentionally located outside the main heating zone^8^. After this process, the thermocouple orifice was welded shut, as we noticed it is one of the most common sources of furnace leak points.

The pressure inside the furnace significantly impacts the position of the gradient zone (Fig. 1a,b). When the pressure is increased, the temperature gradient is smaller, and the temperature at the same location is higher. We also determined the impact of the gas flow, measured as the opening angle of the throttle valve, on the temperature (Fig. 1c, Fig. S2). The temperature changes nonlinearly with the modifications of the gas flow. There is a local temperature minimum at approx. 200–500 sccm. These results show that parameters in CVD are intertwined and cannot be easily separated, as changes in one parameter impact others.

In the next stage of determining the characteristics of the furnace, we investigated the impact of the system setup on the time relationships between different process events, like achieving the set temperatures or the onset of sulfur melting. As an example, we chose the quartz slab which was used to easily load and unload the substrates throughout the experiments, as pictured in Fig. S1c. However, its presence has a considerable impact on the ramping time. As presented in Table 1, the differences can exceed one minute. This one-minute difference might heavily influence the growth thermodynamics and kinetics, and it must be considered when incorporating new elements inside (new sample holders or heat shields) or additional elements outside (external heater with different lengths) the quartz tube. It is often omitted in the research articles, but presenting the time parameters will facilitate the comparison of the results between different groups.

**Table 1:** The time differences with and without a quartz slab inserted in the quartz process tube. The results shown are the averages of the two processes. The process temperature was set to 900 °C, and the sulfur heater temperature to 130 °C.

	Time between turning on furnace heating and furnace reaching the set temperature (mm: ss)	Time between turning on furnace heating and sulfur heater reaching the set temperature (mm: ss)	Time between turning on the furnace heating and sulfur starting to melt (mm: ss)	Time between turning on the furnace heating and turning on the sulfur heating at 700 °C (mm: ss)
With quartz slab	25:10	20:42	23:23	17:05
Without quartz slab	23:50	19:54	23:00	16:12

### Characterization of the grown layers

**Fig. 2:**
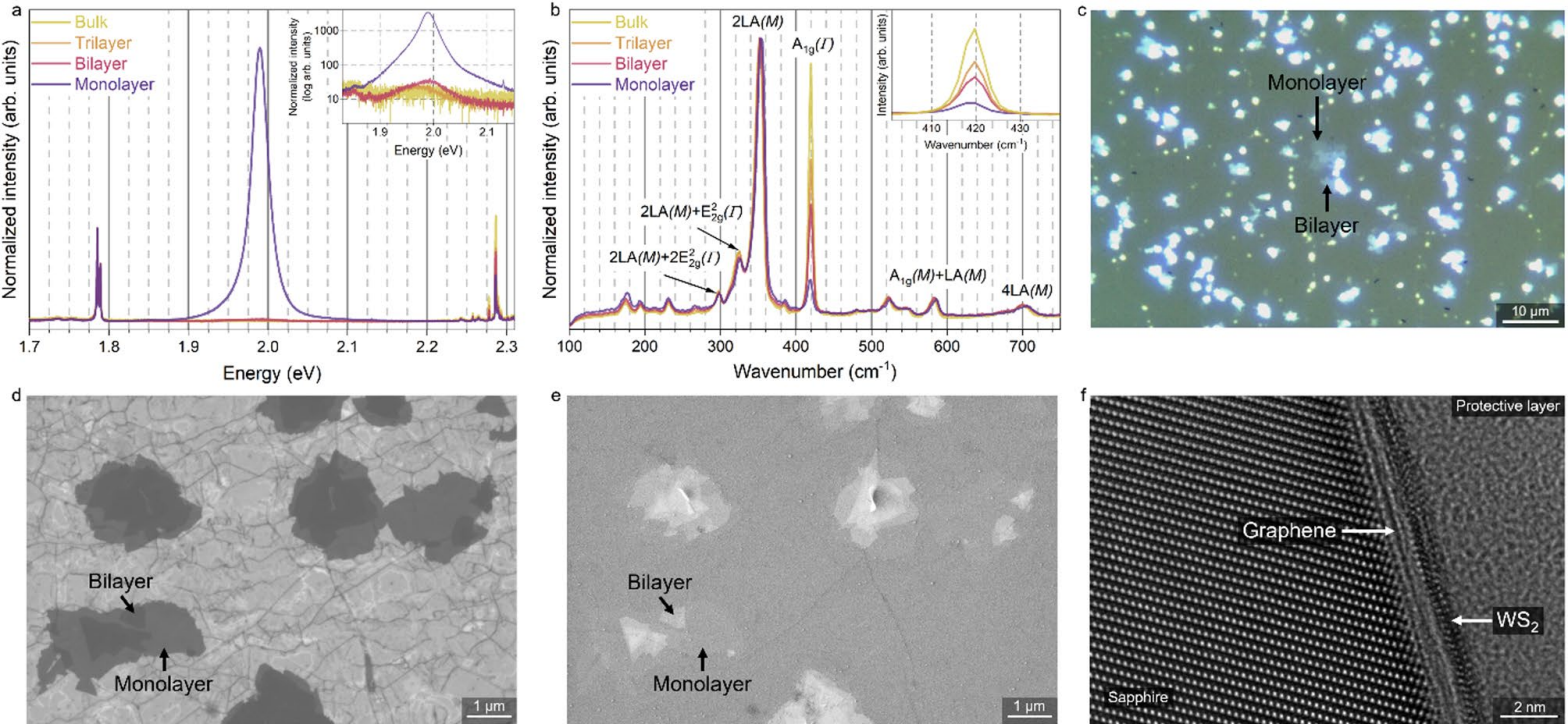
Characterization of WS_2_/graphene vdWHSs, displaying different methods to distinguish the number of layers: (**a**) PL spectra; (**b**) Raman spectra; (**c**) optical micrograph; (**d**,**e**) SEM images collected using in-lens (**d**) and SE (**e**) detectors; (**f**) an HRTEM image of the WS_2_/graphene cross-section.

The main aim of this work was to investigate the growth technology of van der Waals heterostructures. Therefore, we chose WS_2_/graphene vdWHSs as the main 2D system due to the relatively large domains of WS_2_ compared to MoS_2_, which can be easily observed in an optical microscope. As shown in Fig. 2, the WS_2_ monolayers and multilayers can be easily distinguished by the intensity of the PL peak and by the relative intensity of the A_1g_(*Γ*) Raman peak, optical contrast, and SEM imaging using an in-lens detector. The monolayered nature of WS_2_ and graphene is confirmed by transmission electron microscopy (Fig. 2f). Graphene is intact after the growth process, but the intensity of the WS_2_ PL peak causes the graphene peaks to be difficult to distinguish (Fig. S3). The detailed study of the properties of WS_2_/graphene heterostructure is presented in our previous work^24^.

### Influence of flow and pressure on the growth zone

**Fig. 3:**
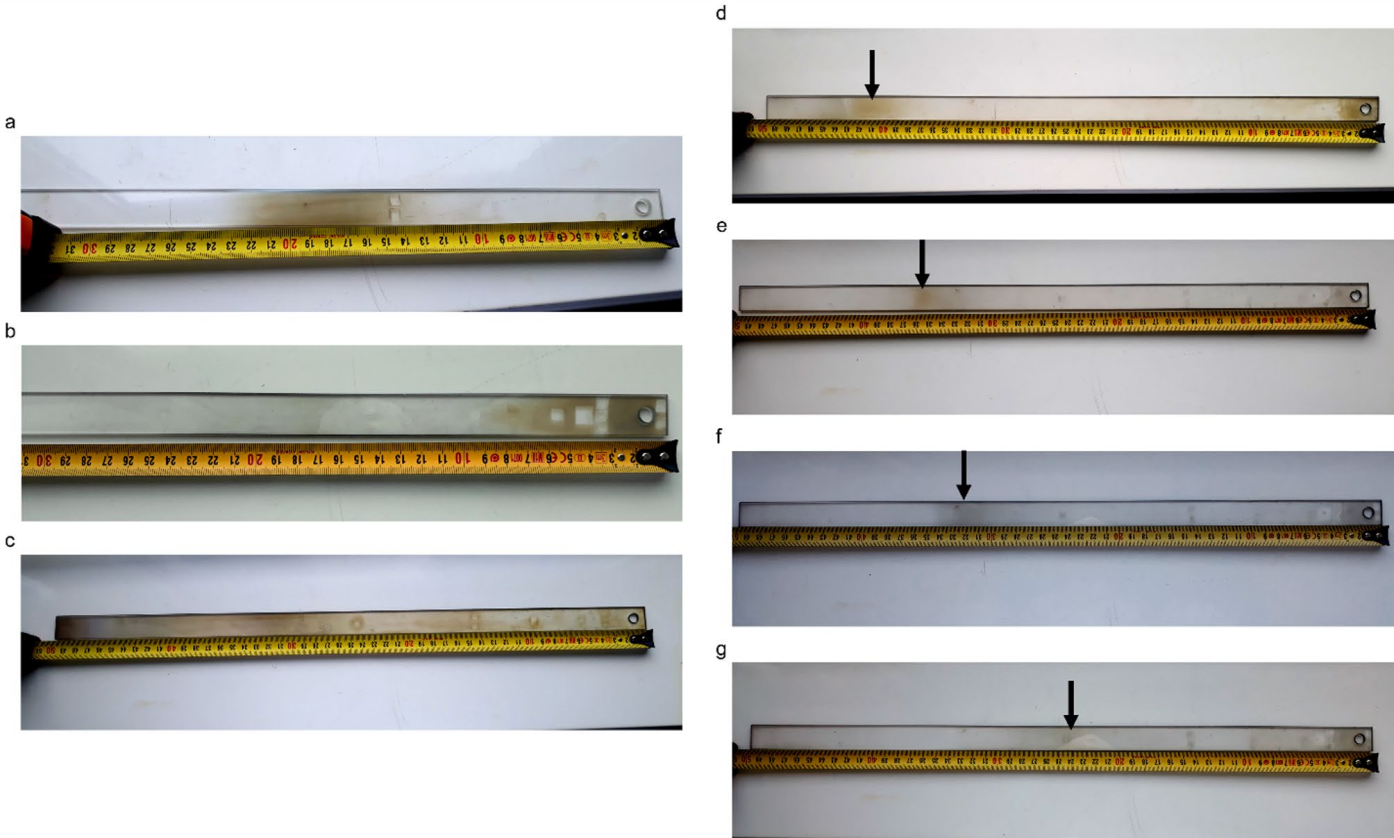
The location of the growth zone depending on the pressure and flow values. The precursors in the boats were located to the right of the quartz slab, upstream. The single growth zone can be located: (**a**) in the center at 30 sccm/950 mbar; (**b**) in the beginning at 500 sccm/950 mbar; (**c**) continuous at 100 sccm/0.3 mbar. The growth zone can be doubled, with one located near the precursors’ boat and secondary (marked with black arrows) located further downstream (**d**–**g**). The secondary zone shifts positions depending on the pressure (at 100 sccm): (**d**) 200 mbar; (**e**) 400 mbar; (**f**) 600 mbar; (**g**) 800 mbar. The contrast of the image was increased to improve the readability.

After establishing the initial growth parameters, we investigated how independent variables affect growth outcomes. We started by modifying the substrates’ location relative to the position of the precursors. There are two main ways of placing the substrates—locating face-down over the metal precursor crucible or face-up downstream, several centimeters away. While the first approach enables the growth of large domains, the samples are extremely non-uniform (Fig. S4). Hence, we placed the substrates downstream of the precursors for this study. Our experiments show that the growth zone is mobile and strongly depends on the process parameters (Fig. 3). For example, at high pressure and low flow, it is approximately at the center of the quartz slab, 220 mm from precursors (Fig. 3a), but it can be near the precursors (Fig. 3b) or stretch across the whole quartz slab (Fig. 3c). Besides flow, pressure also strongly impacts the growth zone, as shown in Fig. 3d–g. Therefore, for our study, we placed several graphene substrates (four or six) on the quartz slab to better understand the WS_2_ synthesis.

### Evaporation of solid precursors

Thermodynamics and kinetics of the CVD synthesis process are heavily dependent on the amount of available volatile precursors^25,26^. Hence, throughout 43 processes, we weighted precursors before and after each process to assess how much vapors were available for the reaction. The selected results are presented in Tables 2 and 3, and the whole dataset is summarized in Table S1. The most notable differences in evaporated WO_3_ are caused by the weight of the added NaCl, which is used as a growth promoter that reacts with WO_3_ and produces volatile oxychlorides^27,28^. The evaporation of precursors depends less on pressure, growth time, and gas flow.

The variations in sulfur evaporation weight are much higher (Table 3). Contrary to WO_3_, evaporation depends strongly on all tested variables, that is, pressure, gas flow, the main growth zone’s temperature, and the S heater’s temperature and length. The most apparent differences are observed when the pressure is changed. Sulfur completely evaporates below a certain pressure threshold, so the vapor flux changes drastically during the growth process, making it unstable over process time. These experiments show that one must account for these parameters when designing the growth experiment and be aware that these variables are inevitably tied together and might vary over the process in this growth method.

**Table 2:** The differences in the evaporated WO_3_ depending on the process parameters. The initial weight of WO_3_ was 200 ± 1.5 mg.

Weight of NaCl (mg)	Furnace temperature (°C)	Pressure (mbar)	Growth time (min)	Ar flow (sccm)	Difference in WO_3_ weight (mg)
10.3	900	950	15	100	20.6
0.0	900	950	15	100	4.8
5.4	900	950	15	100	14.3
20.8	900	950	15	100	44.8
10.6	700	950	15	100	4.3
10.7	800	950	15	100	18.9
10.1	1000	950	15	100	24.5
10.0	1100	950	15	100	24.4
10.1	900	0.3	15	100	22.0
10.3	900	20	15	100	23.4
9.8	900	20	30	100	22.2
10.0	900	20	3	100	23.2
10.0	900	200	15	100	22.4
10.1	900	400	15	100	23.1
10.1	900	600	15	100	21.7
10.1	900	800	15	100	22.2
10.1	900	950	15	30	22.4
10.1	900	950	15	500	21.2
10.0	900	950	15	10,000 (N_2_)	26.0

**Table 3:** The differences in the evaporated S depending on the process parameters. The initial weight of sulfur was 1000 ± 3 mg.

Furnace temperature (°C)	Pressure (mbar)	Growth time (min)	Ar flow (sccm)	S heater temperature (°C)	Sulfur heater start temperature (°C)	Length of sulfur heater (mm)	Difference in S weight (mg)
900	950	15	100	130	700	100	22.2
900	950	15	100	150	700	100	44.8
700	950	15	100	130	550	100	10.1
800	950	15	100	130	600	100	15.8
1000	950	15	100	130	800	100	46.6
1100	950	15	100	130	1000	100	80.1
900	0.3	15	100	130	700	100	1002.3 (all)
900	20	15	100	130	700	100	114.9
900	20	15	100	130	700	220	231.2
900	20	15	100	150	700	220	678.8
900	20	30	100	130	750	220	433.8
900	20	3	100	130	750	220	50.3
900	200	15	100	130	700	100	58.6
900	400	15	100	130	700	100	44.4
900	600	15	100	130	700	100	33.8
900	800	15	100	130	700	100	31.2
900	950	15	30	130	700	100	23.6
900	950	15	500	130	700	100	20.1
900	950	15	10,000 (N_2_)	130	700	100	7.6

### Statistical analysis

Ideally, the outcomes of the growth processes could be predicted by the analysis of the input parameters. However, as it will be discussed in the “Repeatability of the growth process” section, it is very challenging due to the seemingly inherent variability in the CVD growth of vdWHSs. Therefore, we focused on analyzing a more accessible and stable output parameter (dependent variable)—the evaporation of sulfur and tungsten oxide (denoted as E-S and E-WO_3_), introduced in the previous section. The evaporation of precursors is directly connected to their flux and, thus, to the outcomes of the growth process. Along with other thermodynamic and kinetic variables, like temperature, pressure, carrier gas flow, or growth time, these parameters govern the growth of 2D materials.

Hence, we performed a statistical analysis of the influence and significance of the growth parameters (independent variables) on the evaporation of sulfur and WO_3_. In contrast to qualitative observations, as presented in the previous section, the statistical approach provides definitive, quantitative results. In our CVD growth process, we control over 40 process parameters, including the position of substrates, gas flows during each stage, duration of stages, position of quartz elements, etc. Most of these parameters were kept unmodified between processes, but several were changed to achieve different growth results. For the statistical analysis, we considered 11 modified independent variables and analyzed their influence on the evaporation of precursors. These independent variables included: weight of NaCl added to WO_3_ (Var1), weight of WO_3_ (Var2), growth zone temperature (Var3), estimated WO_3_ zone temperature (Var4), sulfur zone temperature (Var5), temperature of the furnace when the sulfur zone heater was turned on (Var6; it was an accessible method of controlling the time when sulfur started to evaporate), pressure (Var7), carrier gas flow (Var8), process time (Var9; the time between the growth zone achieving its set temperature and turning off the heating), process tube position (Var10), and the sulfur heating zone length (Var11).

To achieve the most accurate and repeatable results of the statistical analysis of any parametric experiment, it should comply with the Design of Experiment (DoE). However, we decided not to use DoE. Due to the duration of the growth process (the total time for a single process is around 4–6 h, including preparation time and cleaning), the whole parametric growth experiment would be enormous, as even the classical DoE would involve 2048 (the simplest 2^k^ factorial plan) or 177,147 experiments (3^k^ factorial plan). In such a large parametric environment, it would be beneficial to use compositional, fractional, or other plans^29^ that still require hundreds of processes. Hence, our experiment aimed only at screening the variables, enabling us to statistically determine which independent variables significantly affect the evaporation and, thus, the growth outcomes. The growth processes were planned according to the research standard in the CVD field, that is, one-factor-at-a-time^29^. The variables were modified ad-hoc based on the outcomes of the previous processes.

To answer the question which variables have a statistical impact on the evaporation of precursors, we performed the following analyses: (i) analysis of the scatter plots of variables to eliminate possible outliers; (ii) analysis of correlation between dependent variables (E-WO_3_ and E-S) and independent variables (process parameters Var1–Var11); (iii) analysis of correlation between dependent variables; (iv) analysis of the level of variation (coefficient of variation, CV) of the process parameters to reveal possible quasi-constant variables. We omitted the regression analysis due to the small data sample; however, for larger datasets, it will provide further data for the analysis.

We started with the evaluation of the scatter plots of each variable. Based on it, we decided to remove two processes from the analysis, i.e., a process with a nitrogen flow of 10,000 SCCM (instead of typical argon 30–500 SCCM) and a process with 50 mg of WO_3_ (instead of typical 200 mg), as these values significantly differ from other processes and were marked as outliers. These parameters caused increased evaporation of sulfur (process with 10,000 SCCM N_2_) and decreased evaporation of tungsten oxide (process with 50 mg of WO_3_).

Then, we focused on the analysis of the relevance of these correlations, which was based on two basic dependencies: monotonic linear and monotonic nonlinear (the latter denoted as Spearman’s correlation). In general, quadratic correlation should also be included in the analysis. However, it requires regression analysis, but our data was insufficient for it, so it was omitted from this study.

Depending on their strength, the dependencies discussed below can be described as:^29^ (i) 0.8≤|r|≤1 – strong correlation; (ii) 0.5≤|r|<0.8 – moderate correlation; (iii) 0≤|r|<0.5 – weak correlation. However, it is very common in the literature to find a more detailed classification for the value of the correlation coefficient. What constitutes a satisfactory correlation coefficient depends on the purpose for which it is to be used and the nature of the raw data – generally, the greater the amount of data, the lower the acceptable correlation coefficient. By assessing our data, we adopted the following scale to assess the strength of the correlations:^30^ (i) |r|<0.3 – little if any correlation; (ii) 0.3≤|r|<0.5 – low correlation; (iii) 0.5≤|r|<0.7 – moderate correlation; (iv) 0.7≤|r|<0.9 – strong correlation; (v) |r|≥0.9 – very strong correlation.

**Fig. 4:**
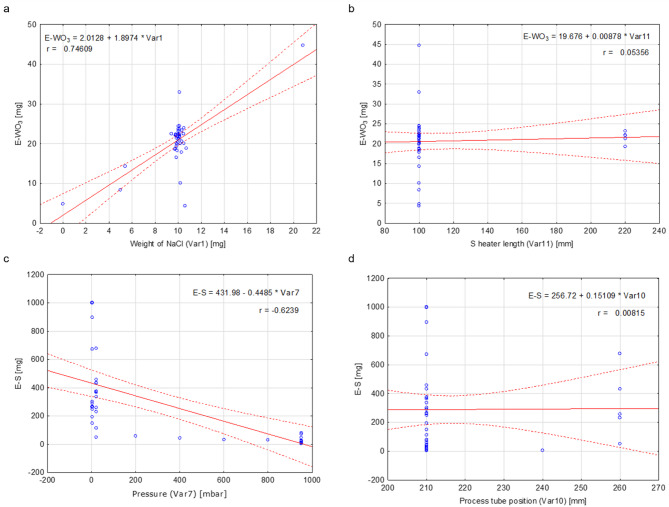
Scatter plots for: (**a**) the highest linear correlation for E–WO_3_, Var1 (weight of NaCl), *r* = 0.746; (**b**) the lowest linear correlation for E-WO_3_, Var11 (S heater length), *r* = 0.054; (**c**) the highest linear correlation for E–S, Var7 (pressure), *r* = 0.624; (**d**) the lowest linear correlation for E–S, Var10 (process tube position), *r* = 0.008.

After removing outliers, we analyzed the monotonic linear and nonlinear correlations between dependent and independent variables. The analysis showed significant differences in linear correlations between independent and dependent variables. For E-WO_3_, the highest linear correlation exists for the weight of NaCl (*r* = 0.746), while the lowest is for the sulfur heating zone length (*r* = 0.054, Fig. 4a,b). Pressure is the highest for E–S (*r* = 0.624), and the process tube position is the lowest (*r* = 0.008, Fig. 4c,d). The complete results of the linear analysis are presented in Table 4. In some cases, the correlation is low or moderate, indicating that a correlation different than linear could exist.

**Table 4:** Table of the linear correlations between dependent and independent variables. The low, moderate, and strong correlations are marked in bold.

	E–S	E–WO_3_	Var1	Var2	Var3	Var4	Var5	Var6	Var7	Var8	Var9	Var10	Var11
E–S	1.000	− 0.021	− 0.057	0.048	0.022	0.032	0.260	− 0.082	**− 0**.**624**	**0**.**358**	0.029	0.008	0.049
E–WO_3_		1.000	**0**.**746**	0.091	**0**.**440**	**0**.**456**	**0**.**325**	**0**.**414**	− 0.084	− 0.267	0.204	− 0.055	0.054
Var1			1.000	− 0.196	0.031	0.044	0.163	0.196	0.015	− 0.044	0.038	− 0.161	0.007
Var2				1.000	0.196	0.212	0.095	0.112	− 0.134	− 0.071	0.046	0.223	0.139
Var3					1.000	**0**.**985**	0.038	**0**.**766**	0.104	**− 0**.**326**	0.275	0.100	0.091
Var4						1.000	0.066	**0**.**764**	0.135	**− 0**.**387**	**0**.**365**	0.113	0.111
Var5							1.000	− 0.188	− 0.239	− 0.265	0.181	− 0.030	− 0.008
Var6								1.000	0.108	− 0.188	0.163	0.141	0.195
Var7									1.000	**− 0**.**379**	**0**.**311**	− 0.186	− 0.255
Var8										1.000	**− 0**.**702**	− 0.294	− 0.269
Var9											1.000	0.241	0.224
Var10												1.000	**0**.**961**
Var11													1.000

**Fig. 5:**
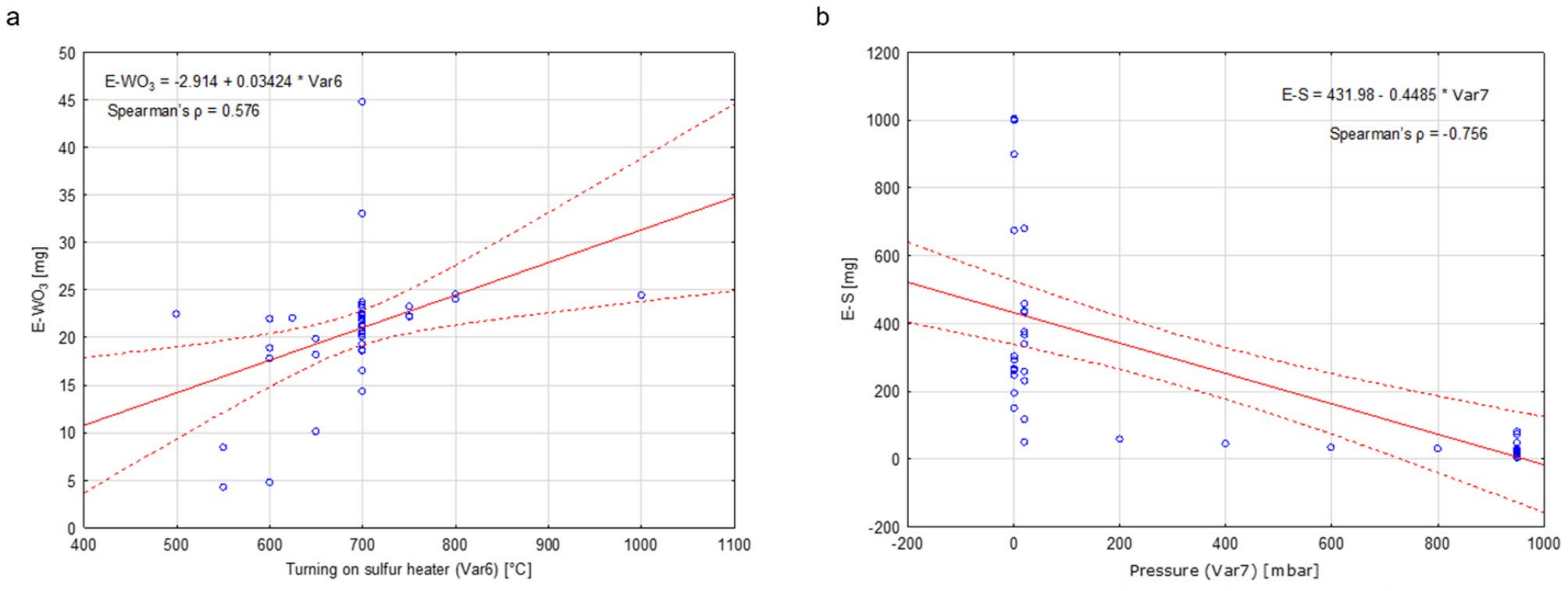
Scatter plots for: (**a**) the highest Spearman correlation for WO_3_ between E–WO_3_ and Var6 (temperature of the furnace when the sulfur zone heater was turned on), Spearman’s *ρ* = 0.576; (**b**) the highest Spearman correlation for sulfur between E–S and Var7 (pressure), Spearman’s *ρ* = − 0.756.

**Fig. 6:**
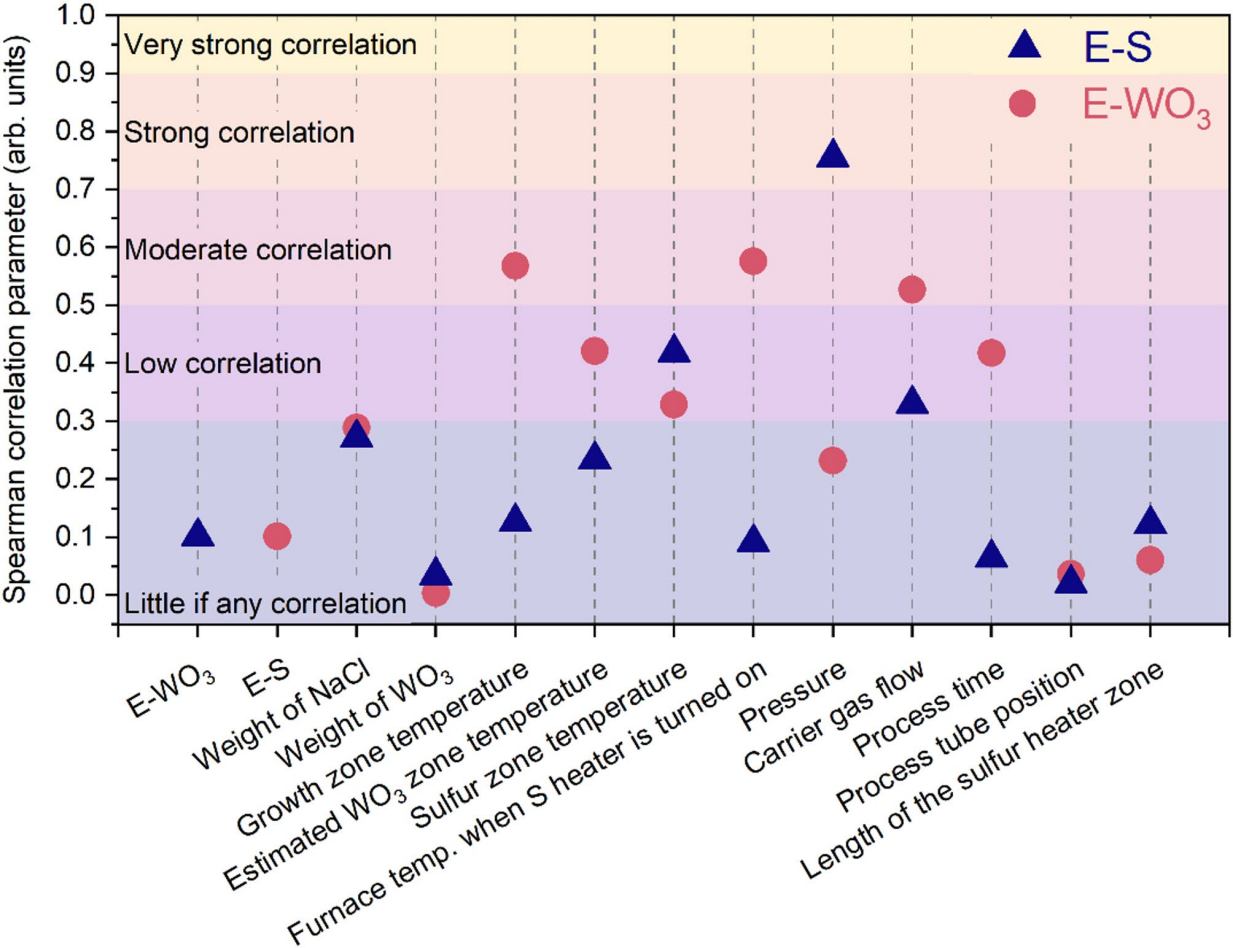
The values of the Spearman correlation parameters between dependent and independent variables.

Spearman correlation coefficient (Spearman’s *ρ*) describes the monotonic nonlinear correlation and can be more versatile as it allows us to determine the power of the monotonic correlation even if it is not linear. The examples of the highest Spearman’s *ρ* are shown in Fig. 5. The highest nonlinear correlation of E–WO_3_ exists with the temperature of the furnace when the sulfur zone heater was turned on and is 0.576 (Fig. 5a). It is closely followed by the growth zone temperature at 0.568. The highest nonlinear correlation for sulfur is with the pressure (Fig. 5b) at − 0.756. The results for the Spearman correlation coefficient between independent and dependent variables are presented in Fig. 6. In the case of Spearman correlations, values are calculated only between the independent and dependent variables, while the relationship between independent variables is determined only based on linear correlation (Table 4).

**Fig. 7:**
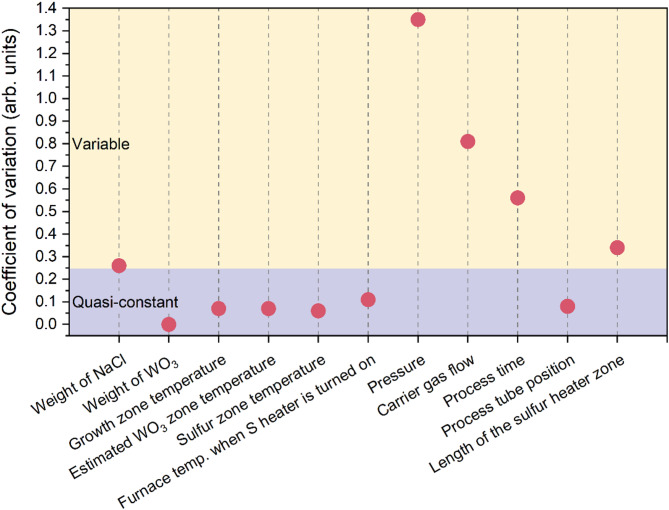
The values of coefficient of variation (CV) for the analyzed independent variables.

In Fig. 7, we showed the values of the coefficient of variation (CV), which is used to evaluate the variability of each independent variable and determine whether they should be considered quasi-constants. In the literature, there are no strict guidelines regarding the threshold above which the variable should be treated as quasi-constant, and no specific value for a coefficient of variation is considered a proper value. Most commonly, the values below 0.1–0.25 are proposed. In this work, we selected 0.25, which means that Var3, Var4, Var5, Var6, and Var10 can be treated as quasi-constants and have no statistical significance to the precursor evaporation. Importantly, this is true only for our analysis and the chosen growth parameters, and these variables might impact the growth process in a different experimental environment. Var2, after removing the outlier, is a process constant and has not been further analyzed.

The presented screening allows us to evaluate which independent variables are significant and should be controlled well during the growth processes. Based on these evaluations, we can draw several conclusions that offer guidelines for future experiments and the 2D materials growth community.

Var1 (weight of NaCl), despite the low variability, is strongly correlated with the evaporation of WO_3_ and influences the growth process. It is expected, as NaCl reacts with WO_3_ at much lower temperatures than the melting point of WO_3_, as discussed earlier. Var2 (weight of WO_3_) is a process constant after removal of the outlier, and its impact on evaporation cannot be determined. Still, the weight of metal precursor is expected to affect the evaporation, but to a lesser extent than NaCl. Nonetheless, by adding NaCl above a certain threshold, the weight of WO_3_ will become the limiting step, as all of WO_3_ will react with the growth promoter.

Var3 (growth zone temperature) has low variability but shows a moderate correlation with E–WO_3_ and should be investigated further. In particular, the temperature of the growth zone impacts the thermodynamics of the crystal synthesis rather than the evaporation of precursors. Var4 (estimated WO_3_ zone temperature) is strongly correlated with Var3; in our experiment, it also has a moderate impact on evaporation.

Var5 (sulfur zone temperature) is weakly (for E–WO_3_) or moderately (for E–S) correlated with the dependent variables, and its variability is low due to the selected process parameters. Hence, its impact on the process outcomes cannot be determined based solely on the statistical analysis, but the sulfur zone temperature is expected to impact the sulfur evaporation rate. Var6 (temperature of the furnace when the sulfur zone heater was turned on) has low variability, although it is moderately correlated with the evaporation of WO_3_. Interestingly, its combined linear and nonlinear correlation is the highest among other variables. This result is surprising, as it was expected to affect sulfur, not WO_3_, evaporation. It can probably be explained by the fact that Var6 is strongly correlated with the growth zone and estimated WO_3_ zone temperatures and the selected growth parameters caused the observed influence.

Var7 (pressure) is the variable that has the highest correlation with sulfur evaporation. We unambiguously claim that this variable strongly impacts sulfur evaporation and should be closely monitored during the growth of vdWHSs. Var8 (carrier gas flow) has the highest variability among all tested variables. However, the gas flow itself must be considered in connection to the geometry of the furnace. If the reactor chamber diameter is higher, it will decrease the linear velocity of the gas, which modifies the stagnant layer thickness^31^ and impacts the concentration of precursors. Therefore, the gas flow values must be chosen for each reactor separately. On a similar note, pressure also impacts the linear velocity of the gas.

Var9 (process time) is moderately variable and shows moderate correlation only with E–WO_3_. Like Var8 (carrier gas flow), the process time cannot be considered without analysing the reactor geometry, as it is only an indirect measure of the total time the precursors have an opportunity to deposit onto the substrate, which is one of the strongest disadvantages of solid-source CVD. Var10 (process tube positions) and Var11 (length of the sulfur heater zone) are correlated and quasi-constants, and they do not affect the evaporation rate of the precursors.

However, the most important conclusion arises when we consider the correlation between both dependent variables, i.e., evaporation of S and WO_3_—they are not correlated at all. As discussed earlier, the flux of precursors critically impacts the growth process. If these two parameters are not correlated, and there are various correlations between independent and dependent variables, it is extremely difficult to control the growth process well. Changing one independent variable can impact either both or only one of the dependent variables, making the analysis of the growth outcomes much more difficult. Thus, one needs to perform many more processes to indicate how the change in the independent variable impacts the growth of vdWHSs.

Based on our screening analysis, we can divide the independent variables into two groups. The first encompasses variables that do not show correlations with the dependent variables. These are: Var10 (process tube position) and Var11 (the sulfur heating zone length). While Var11 shows variability and does not impact the evaporation of precursors, we cannot exclude the impact of Var11, as it was quasi-constant in our screening. The other parameters, that is, Var1 (weight of NaCl), Var3 (growth zone temperature), Var4 (estimated WO_3_ zone temperature), Var5 (sulfur zone temperature), Var6 (temperature of the furnace when the sulfur zone heater was turned on), Var7 (pressure), Var8 (carrier gas flow), and Var9 (process time) show at least moderate impact, either linear or nonlinear, on the evaporation of the precursors. These parameters must be monitored closely during growth to achieve highly reproducible results. Additionally, our analysis showed the complexity and problems associated with the statistical analysis of such complex experiments as CVD growth of 2D materials and provided a guideline for approaching the design of experiment for these processes.

### Repeatability of the growth process

As a final experiment, we performed five identical, consecutive growth processes to assess the repeatability of the solid-source CVD method. Contrary to the previous sections, we focused on the growth of the WS_2_ crystals instead of the evaporation of precursors. We did not air-bake the quartz tube between the growth runs because it caused a significant electrical charge of the quartz elements, resulting in the precursors particles escaping the quartz crucibles and the instability of the weighing scale. Also, as shown in other works, charged substrates modify the growth process outcomes^32,33^. After the synthesis process, the quartz slab and crucibles were cleaned mechanically and with acetone to remove any remaining precursors. The furnace was operated by a single person to minimize the operator-induced differences between processes. We placed the substrates and crucibles with ± 1 mm accuracy, and the evaporation area of the precursors was also kept ± 1 mm^2^ between processes. In this experiment, the average weight of NaCl, WO_3_, and S was 10.06 ± 0.08, 199.74 ± 0.21, and 1000.67 ± 1.18 mg, respectively. All changes during the growth procedures, like modifications of the gas flow, were applied within 3 s.

As can be seen in Table 7, the dependent variables are very similar. The standard deviation of WO_3_ and S evaporation weight differences is 0.23 and 1.18 mg, respectively, which is a very low value, corresponding to ± 1.5%. The time deviations are also low, ranging from 7 to 13 s (± 1%).

**Table 7:** Weight and time differences between 5 consecutive growth processes with identical growth conditions.

	Difference in WO_3_ weight (mg)	Difference in S weight (mg)	Time between turning on furnace heating and furnace reaching set temperature (mm: ss)	Time between turning on furnace heating and sulfur heater reaching set temperature (mm: ss)	Time between turning on furnace heating and sulfur melting (mm: ss)	Time between turning on furnace heating and turning of sulfur heating (mm: ss)	Time to cool furnace from 900 to 700 °C (mm: ss)
Process 1	23.33	76.28	23:28	19:31	22:16	16:12	54:32
Process 2	23.83	74.35	23:48	19:46	22:46	16:22	54:55
Process 3	23.88	74.37	23:55	19:47	22:40	16:27	55:05
Process 4	23.74	72.71	23:25	19:35	22:14	16:11	54:34
Process 5	24.02	75.27	23:50	19:45	22:32	16:24	54:58
Standard deviation	0.23	1.18	00:12	00:07	00:13	00:07	00:13

**Fig. 8:**
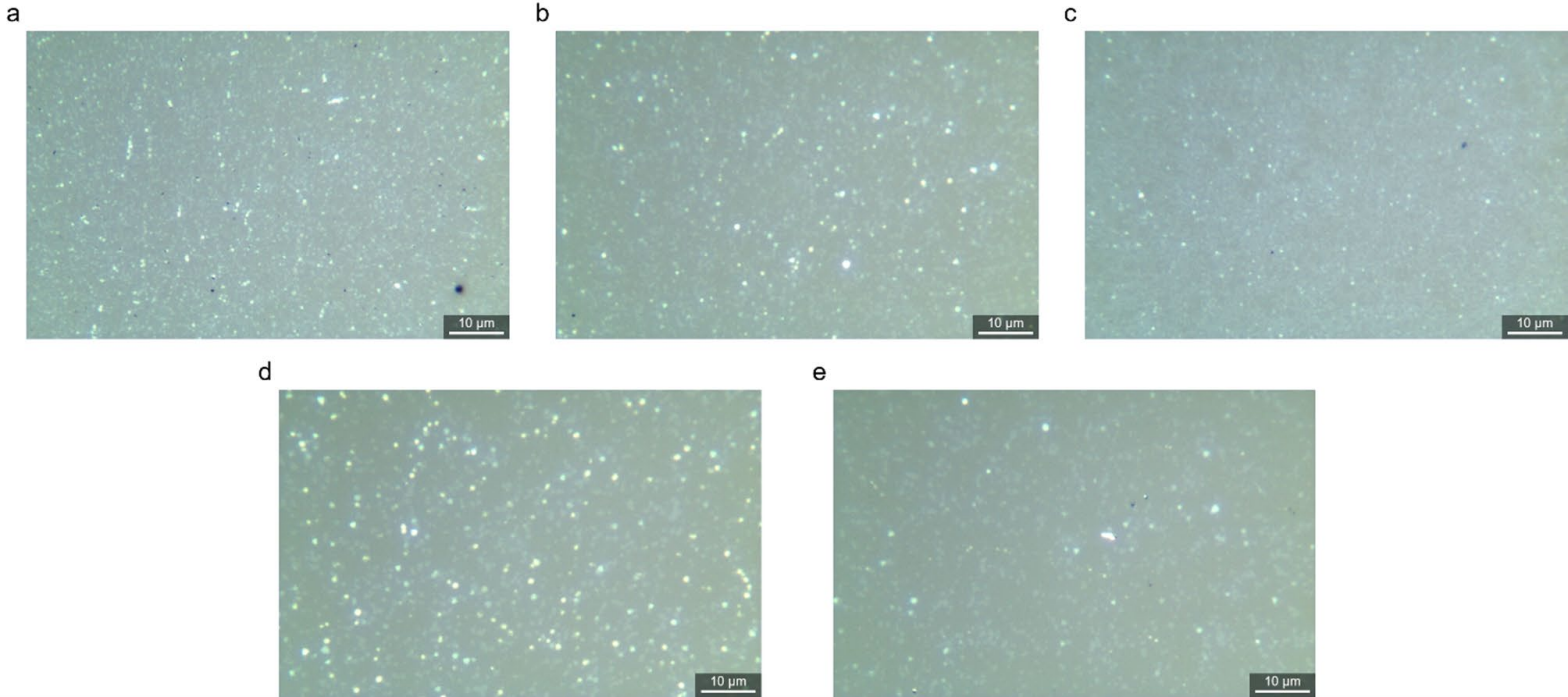
Optical micrographs of WS_2_/graphene samples located 72 mm from the center of WO_3_ powder. Samples a-e were grown in five identical, consecutive growth processes.

We characterized the resulting samples, focusing particularly on the morphology of WS_2_ crystals. For this purpose, we captured optical images using a Raman/PL microscope with ×﻿1000 magnification in the centers of the samples to exclude sample edge effects and to avoid any macroscopic defects, such as scratches. Simultaneously, we determined the number of layers using the intensity of PL and Raman spectra. As shown in Fig. 8, there are notable differences between growth runs at the default position of substrates, that is, 72 mm from the WO_3_ powder. For example, the sample in Fig. 6c is covered with a nearly continuous monolayer of WS_2_ (which is visible as white halo), while in the sample shown in Fig. 6e, the monolayer coverage is significantly lower. The coverage of multilayers also varies. The optical micrographs of substrates located at 42, 182, and 282 mm from the WO_3_ can be found in Figs. S5–S7, where the differences are even more pronounced.

**Fig. 9:**
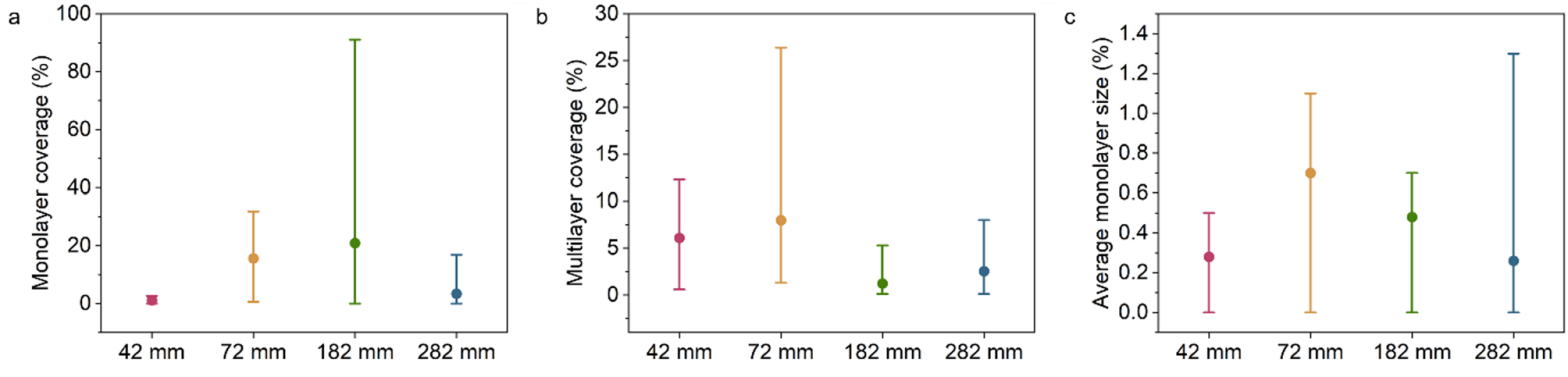
The summary of the independent variables in 5 consecutive processes.

We analyzed these images using the ilastik package. We distinguished monolayers from multilayers based on the optical contrast and PL spectra and segmented the images accordingly. Then, using ImageJ software, we calculated the coverage of monolayers and multilayers and the average monolayer size. The results, sorted via the distance from WO_3_ precursors, are summarized in Fig. 9. The first process differs starkly from the others at each distance, and there are notable variations between processes 2–5, as confirmed by high standard deviations in monolayer coverage. It shows that even though the parameters are well controlled and the evaporation of precursors is very similar between processes, there is an inherent variability in process repeatability of the solid-source CVD growth of van der Waals heterostructures.

In addition to this repeatability study, we also performed a few experiments in which we did two of the same processes of MoS_2_ and WS_2_ growth on graphene. The results can be found in Fig. S8. Some of the differences are even more significant than shown in Fig. 6, resulting in completely different morphologies. This observation confirms that the variability is not limited to WS_2_/graphene but applies to MoS_2_/graphene.

We cannot pinpoint the single cause of the observed variations, although several hypotheses arise. First, we monitored the furnace leak by pumping it to 50 mbar and observing the pressure changes for 10 min. In this experiment, the average change was 0.2 mbar, but it is close to the accuracy of the used pressure sensor (0.1 mbar). The more accurate pressure change was measured overnight and was ~ 1.5 mbar, translating to approx. 0.3 mbar·L/h or 8 × 10^− 5^ mbar L/s. A system with this leak rate is much tighter than a commercial-grade CVD system^34^ and described as a high vacuum system with a leak by the DESY synchrotron report^35^. Air inflow may interfere with the growth process, and the acceptable leak in 2D materials growth must be even lower. In particular, a recent report shows that the presence of oxygen in the system is one of the critical parameters impacting the reproducibility of 2D growth^36^. The airtightness argument is valid both for LPCVD and APCVD, although it is much more prominent in LPCVD. While the gas diffusion will be much higher in the LPCVD system due to the more pronounced leak, Fick’s diffusion is still present in APCVD, and atmospheric pressure reactors should remain airtight to ensure reproducibility.

Another explanation is based on the variability of the substrate. We used graphene on sapphire, and despite the research efforts^21,37^, this material is microscopically imperfect regardless of the high wafer-to-wafer uniformity (Fig. S9). Graphene wrinkles are the most common mesoscopic defects (Fig. S9b), and there is also a moderate D peak (Fig. S9c), which indicates microscopic imperfections. Raman mapping shows G and 2D peak position variability, indicating strain and doping variances (Fig. S9d). In our previous study on the selective growth on electron-beam irradiated graphene^32^, we showed that the greater defect density in the graphene substrate yields a noticeable enhancement in both coverage and domain size of monolayer WS_2_. The relative nucleation decreased from 0.247 to 0.013 domain/µm^2^, while coverage rose from 2.5 to 26.6% and the average domain size from 2.3 to 21.5 µm^2^ when the graphene D peak area increased from 3200 to 7900 arb. units. These results support our claim that even minor variations in the defect density in the graphene substrate can lead to significant changes in the morphology of the synthesized TMDs. Therefore, for future studies, it is advised to use graphene without microscopic defects, like epitaxial graphene grown on ultra-flat Cu(111)^38^ or wide-step SiC^39^.

## Conclusions

In summary, we investigated the technology of solid-source CVD of van der Waals heterostructures. We showed that the growth parameters in this method are intertwined and cannot be separated. For example, changing the pressure results in the modification of sulfur vapor flux and shifts the growth zones, modifying the process’ thermodynamics and kinetics. Hence, we advocate using methods that enable much better degree of control. One these methods is MOCVD, in which the precursors (typically liquids or high-vapor-pressure solids, like tungsten hexacarbonyl) are stored in separate containers, and their flux can be controlled independently of the process parameters.

The statistical screening of the process variables showed that most parameters have a moderate or significant impact on the evaporation of precursors. We indicated how the statistical analysis of 2D materials could be performed to quantitatively assess the impact of the growth variables. Importantly, the proposed analysis can be done regardless of growth method or modification, including MOCVD.

Our repeatability studies showed that the solid-source CVD growth of vdWHSs is highly unstable. We discussed the possible causes of growth instability: the limited airtightness of CVD systems and the variances in the graphene substrate surface. We suggest that for improved results, the CVD system must provide a leak rate much better than 8 × 10^− 5^ mbar·L/s. Additionally, the substrate used in the growth of van der Waals heterostructures should exhibit minimal defects that serve as undesired nucleation spots.

Finally, we encourage to report all experimental details of their processes, like the weight of evaporated precursors or the leak rate, as it will increase the reproducibility between different groups and accelerate the development of 2D materials growth technology.

## Supplementary Information

Below is the link to the electronic supplementary material.

Supplementary Material 1

## Data Availability

All relevant data is available upon request from the authors and/or included in the Supplementary Information files. For data requests, please contact the corresponding author (jakub.sitek@pw.edu.pl).
