# Supplementary material for: Technological limitations of solid-source chemical vapor deposition of van der Waals heterostructures
Source: Sci Rep. 2025 Aug 5;15:28517. doi: 10.1038/s41598-025-13921-4 (PMC12325618; doi:10.1038/s41598-025-13921-4)
Supplement: Supplementary file 1 — Supplementary Material 1 [file 41598_2025_13921_MOESM1_ESM.docx]

**Supporting information to:**

Technological limitations of solid-source chemical vapor deposition of van der Waals heterostructures

Jakub Sitek,^1,2,3*^, Wojciech Sitek^4^, Ben R. Conran^5^, Xiaochen Wang^5^, Clifford McAleese^5^, Anna Kaleta^6^, Sławomir Kret^6^, Iwona Pasternak^1^, Mariusz Zdrojek^1^, Włodek Strupiński^1^

^1^ Faculty of Physics, Warsaw University of Technology, Koszykowa 75, 00-662 Warsaw, Poland

^2^ Center for Multidimensional Carbon Materials, Institute for Basic Science, UNIST-gil 50, 44919 Ulsan, South Korea

^3^ Center for Terahertz Research and Applications CENTERA2, Warsaw University of Technology, Poleczki 19, 02-822 Warsaw Poland

^4^ Faculty of Mechanical Engineering, Silesian University of Technology, Konarskiego 18A, 44-100 Gliwice, Poland

^5^ AIXTRON Ltd, Buckingway Business Park, Anderson Road, Swavesey, Cambridge, CB24 4FQ, United Kingdom

^6^ Institute of Physics, Polish Academy of Sciences, Al. Lotników 32/46, 02-668 Warsaw, Poland

*email: jakub.sitek@pw.edu.pl


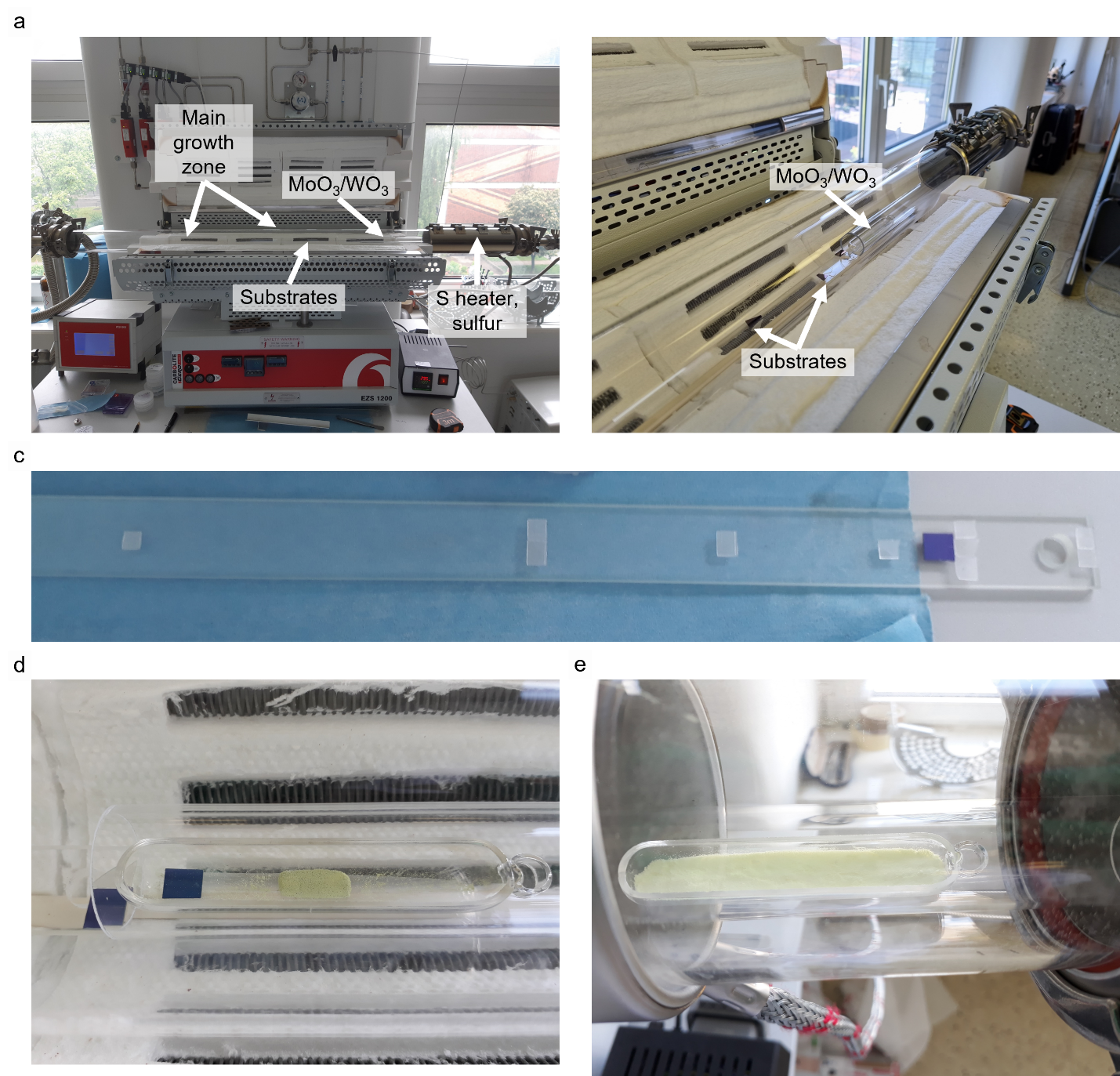


Figure S1. Photographs of the system used in the study: (a) overview of the system; (b) photograph of the arrangement inside the quartz process tube; (c) photograph of the substrate positioning; (d, e) images of the location and alignment of the precursors.


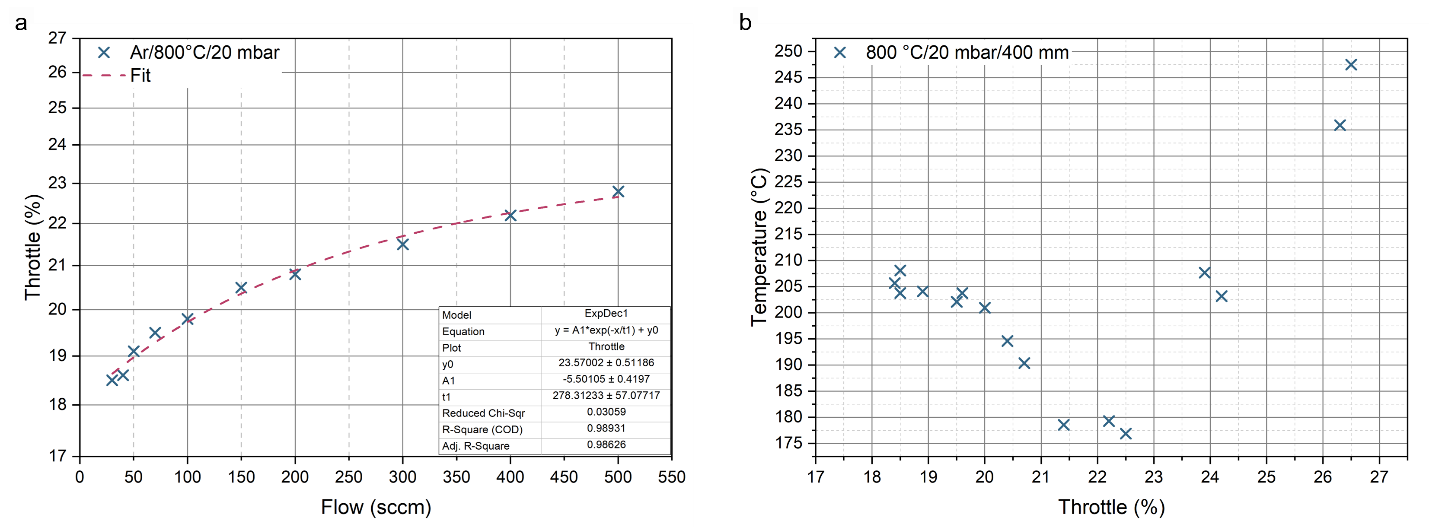


Figure S2. Data used to recalculate flow values in the system: (a) the actual flow-throttle dependence; (b) the actual throttle-temperature dependence.


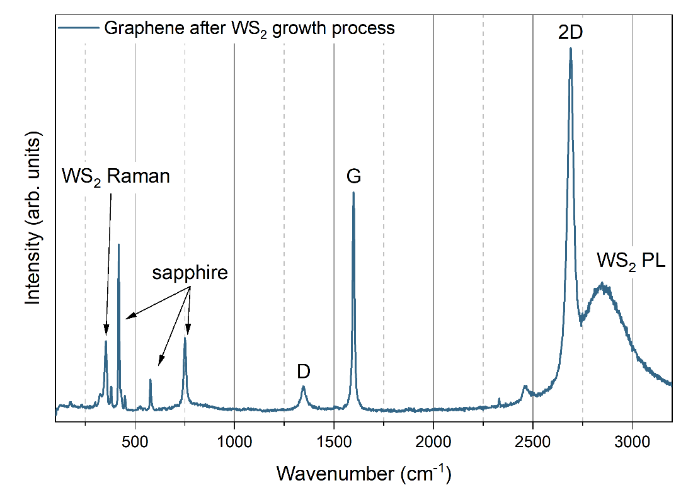


Figure S3. Raman spectrum of graphene after WS_2_ growth, with low WS_2_ photoluminescence intensity.


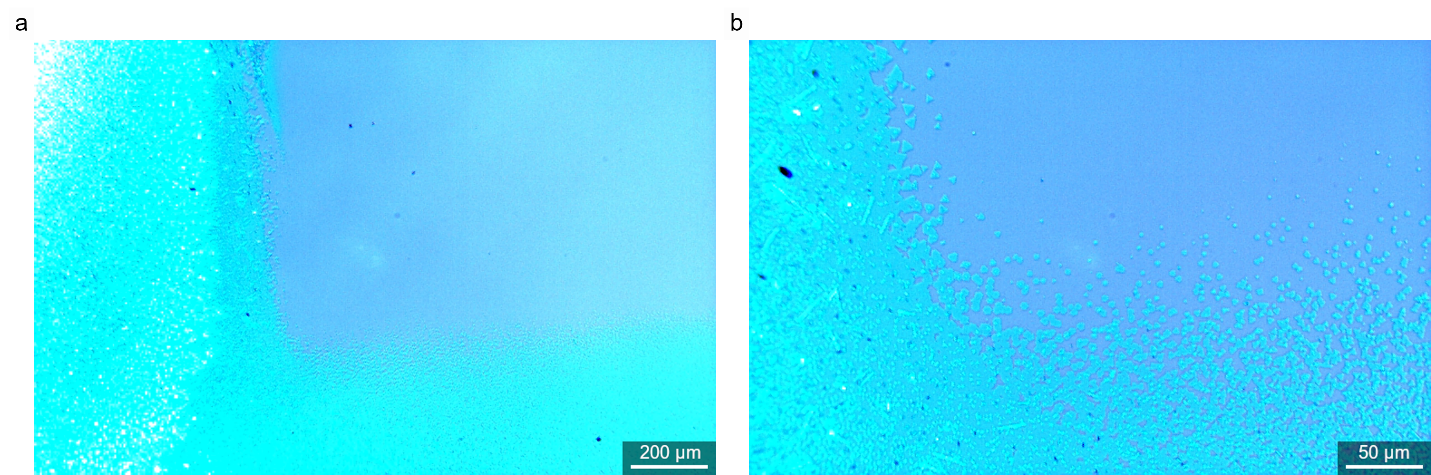


Figure S4. The optical micrographs showing the nonuniformity of MoS_2_ synthesized on SiO_2_ substrate that was place face-down over the precursor crucible.


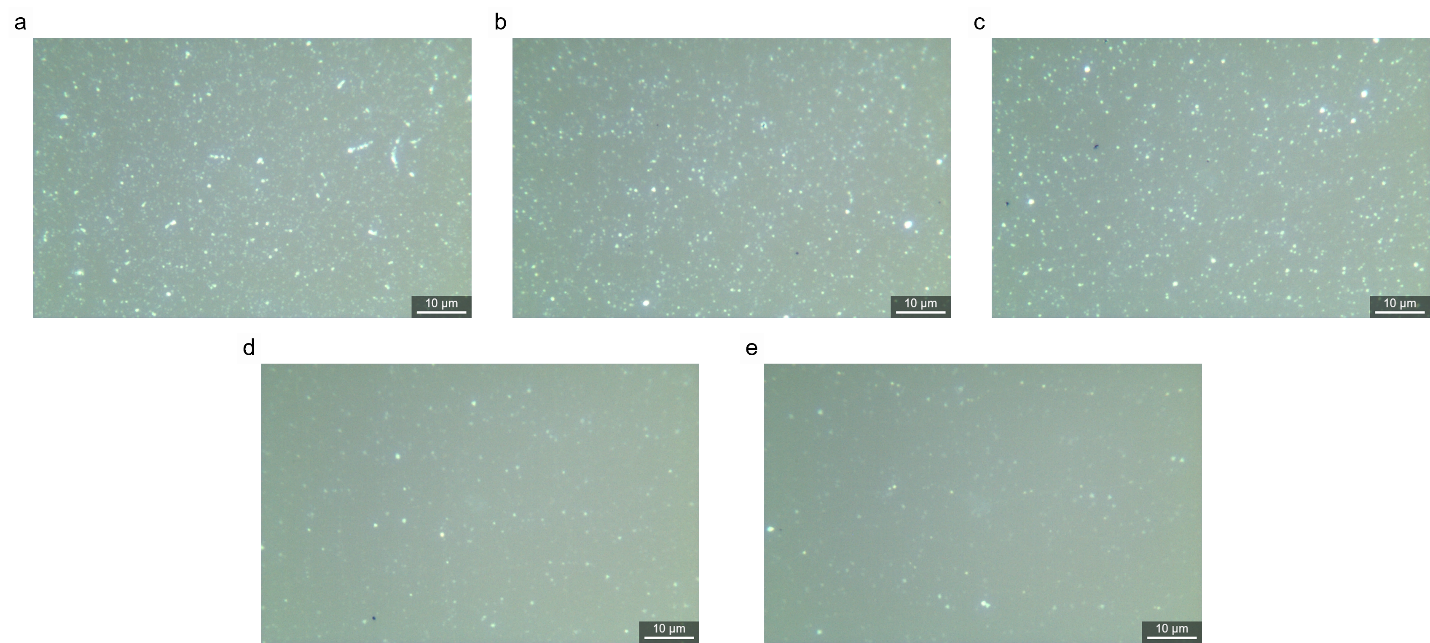


Figure S5. Optical micrographs of WS_2_/graphene samples located 42 mm from the center of WO_3_ powder. Samples a-e were grown in five identical, consecutive growth processes.


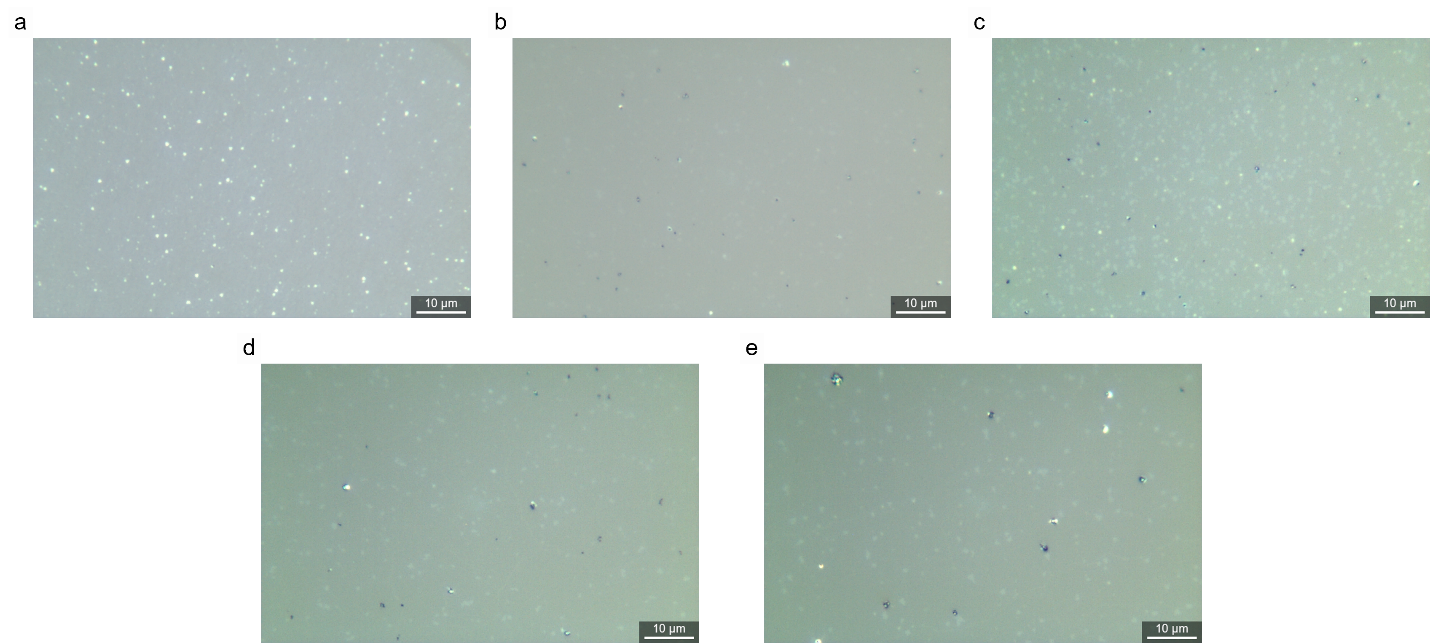


Figure S6. Optical micrographs of WS_2_/graphene samples located 182 mm from the center of WO_3_ powder. Samples a-e were grown in five identical, consecutive growth processes.


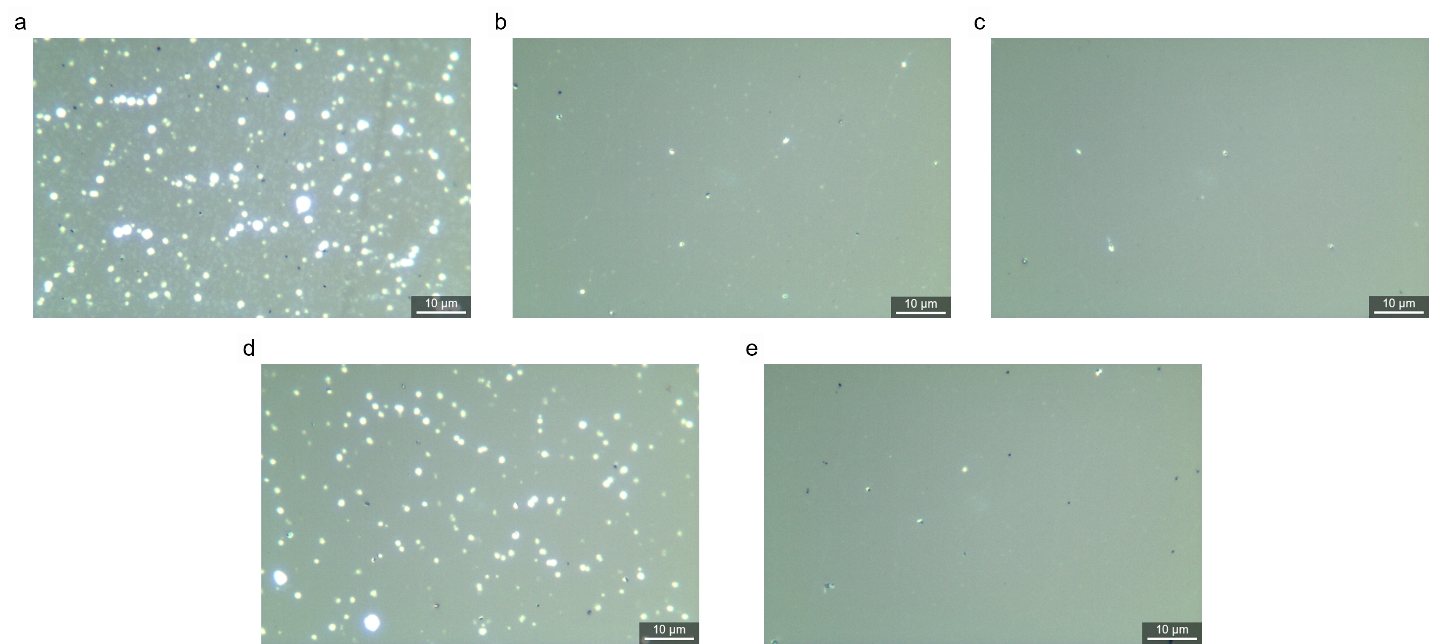


Figure S7. Optical micrographs of WS_2_/graphene samples located 282 mm from the center of WO_3_ powder. Samples a-e were grown in five identical, consecutive growth processes.


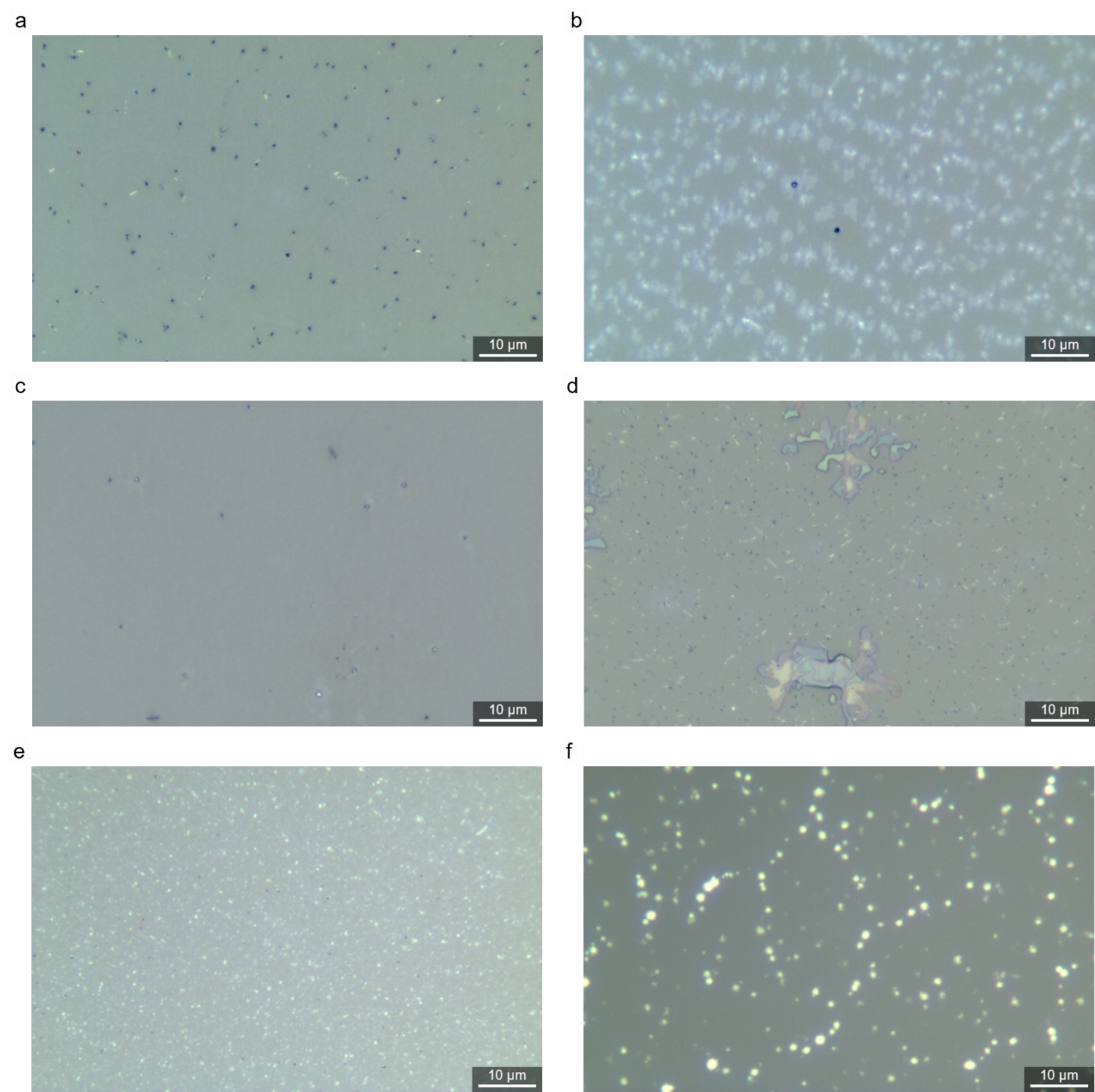


Figure S8. The optical micrographs of pairs of the results of separate growths with identical growth parameters. Figures (a-d) shows two pairs of MoS_2_/graphene process outcomes, and (e-f) shows a pair of WS_2_/graphene samples.


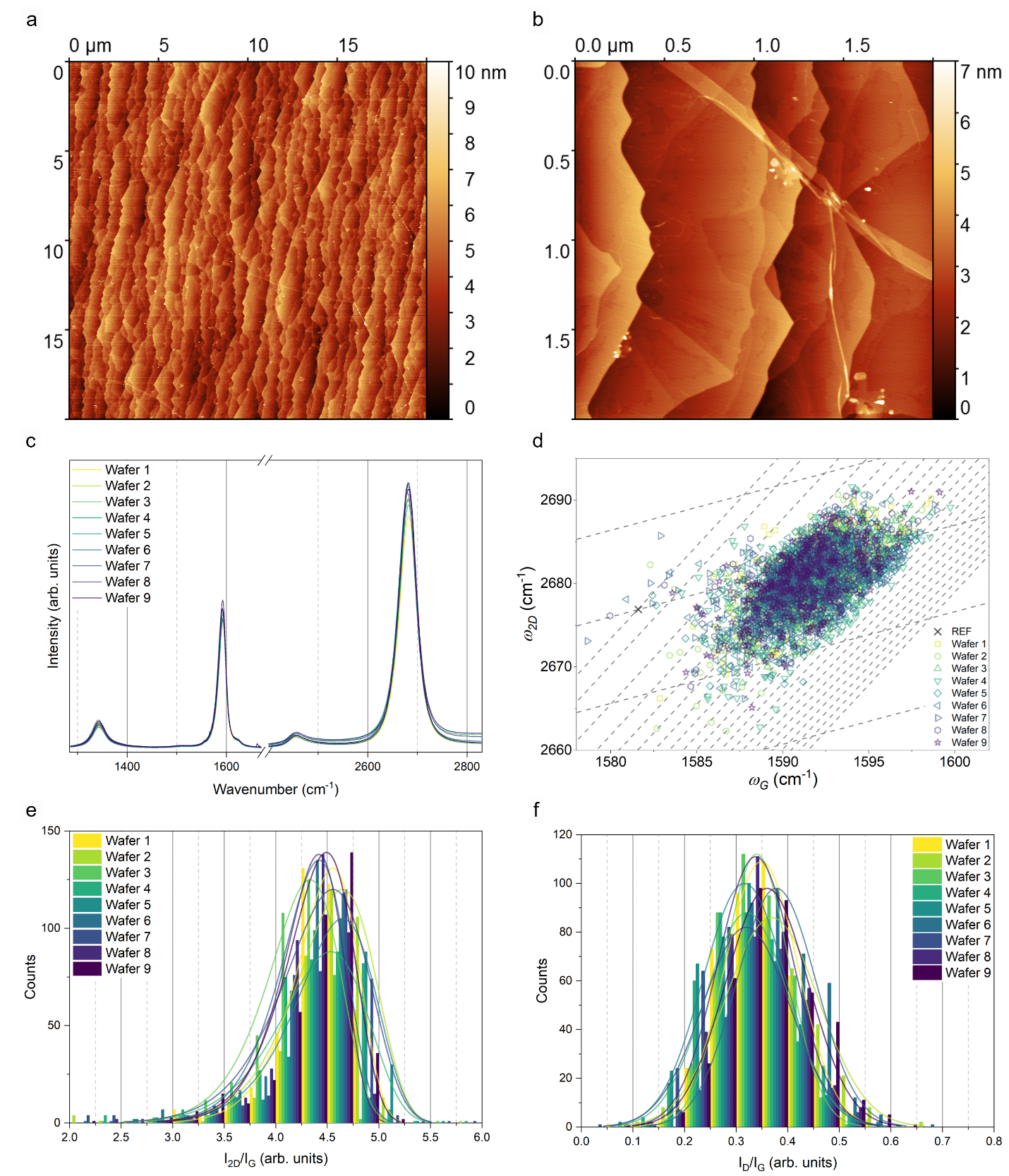


Figure S9. Defects and nonuniformity of CVD graphene on sapphire: (a, b) AFM images showing the graphene wrinkles and sapphire atomic steps that might serve as nucleation sites; (c) the averages of 400 Raman spectra of nine Gr/sapphire 2-inch wafers, grown in a single growth run; (d) strain-doping graph of nine Gr/sapphire 2-inch wafers, grown in a single growth run; (e, f) histograms of the ratio of the integrated intensity of 2D and G (e) and D and G peaks (f).

Table S1. The results of the experiments presented in this study. The table shows the most important independent variables and the weight of the evaporated precursors as independent variables.

| Process | Evaporated WO_3_ [mg] | Evaporated sulfur [mg] | Weight of NaCl [mg] | Weight of WO_3_ [mg] | Furnace temperature [°C] | Estimated WO_3_ temperature (based on position) [°C] | S heater temperature [°C] | Turning on sulfur heater [°C] | Pressure [mbar] | Total gas flow [sccm] | Process time [min] | Process tube position [mm] | S heater length [mm] | Rapid cooling |
| --- | --- | --- | --- | --- | --- | --- | --- | --- | --- | --- | --- | --- | --- | --- |
| 180 | **4.8** | **5.5** | 0.0 | 201.3 | 900 | 872 | 130 | 600 | 950 | 100 | 15 | 240 | 100 | no |
| 181 | **14.3** | **22.2** | 5.4 | 198.6 | 900 | 885 | 130 | 700 | 950 | 100 | 15 | 210 | 100 | no |
| 182 | **44.8** | **72.9** | 20.8 | 199.7 | 900 | 884 | 150 | 700 | 950 | 100 | 15 | 210 | 100 | no |
| 183 | **20.6** | **26.1** | 10.3 | 201.1 | 900 | 884 | 130 | 700 | 950 | 100 | 15 | 210 | 100 | no |
| 184 | **4.3** | **10.1** | 10.6 | 197.5 | 700 | 689 | 130 | 550 | 950 | 100 | 15 | 210 | 100 | no |
| 185 | **18.9** | **15.8** | 10.7 | 200.0 | 800 | 786 | 130 | 600 | 950 | 100 | 15 | 210 | 100 | no |
| 186 | **24.5** | **46.6** | 10.1 | 200.0 | 1000 | 981 | 130 | 800 | 950 | 100 | 15 | 210 | 100 | no |
| 187 | **24.4** | **80.1** | 10.0 | 199.7 | 1100 | 1079 | 130 | 1000 | 950 | 100 | 15 | 210 | 100 | no |
| 188 | **22.4** | **23.6** | 10.1 | 200.2 | 900 | 884 | 130 | 700 | 950 | 30 | 15 | 210 | 100 | no |
| 189 | **23.7** | **27.3** | 10.0 | 199.7 | 900 | 884 | 130 | 700 | 950 | 30 | 15 | 210 | 100 | no |
| 190 | **21.2** | **20.1** | 10.1 | 199.3 | 900 | 884 | 130 | 700 | 950 | 500 | 15 | 210 | 100 | no |
| 191 | **26** | **7.6** | 10.0 | 199.6 | 900 | 884 | 130 | 700 | 950 | 10000 | 15 | 210 | 100 | no |
| 192 | **23.4** | **114.9** | 10.3 | 200.1 | 900 | 884 | 130 | 700 | 20 | 100 | 15 | 210 | 100 | no |
| 193 | **22.4** | **58.6** | 10.0 | 199.2 | 900 | 884 | 130 | 700 | 200 | 100 | 15 | 210 | 100 | no |
| 194 | **23.1** | **44.4** | 10.1 | 200.5 | 900 | 884 | 130 | 700 | 400 | 100 | 15 | 210 | 100 | no |
| 195 | **21.7** | **33.8** | 10.1 | 200.6 | 900 | 884 | 130 | 700 | 600 | 100 | 15 | 210 | 100 | no |
| 196 | **22.2** | **31.2** | 10.0 | 199.7 | 900 | 884 | 130 | 700 | 800 | 100 | 15 | 210 | 100 | no |
| 197 | **22** | **1002.1** | 10.1 | 199.6 | 900 | 884 | 130 | 700 | 0.3 | 100 | 15 | 210 | 100 | no |
| 198 | **22.5** | **375.3** | 9.4 | 200.6 | 900 | 884 | 150 | 700 | 20 | 100 | 15 | 210 | 100 | no |
| 199 | **24** | **336.8** | 10.5 | 201.0 | 900 | 884 | 150 | 800 | 20 | 100 | 15 | 210 | 100 | no |
| 200 | **22.4** | **366.3** | 9.8 | 200.1 | 900 | 884 | 150 | 500 | 20 | 100 | 15 | 210 | 100 | no |
| 201 | **21.9** | **457.4** | 9.9 | 199.5 | 900 | 884 | 150 | 600 | 20 | 100 | 15 | 210 | 100 | no |
| 202 | **33** | **372.9** | 10.1 | 199.5 | 900 | 884 | 150 | 700 | 20 | 100 | 15 | 210 | 100 | no |
| 203 | **23.7** | **432.1** | 10.1 | 199.4 | 900 | 884 | 150 | 700 | 20 | 100 | 15 | 210 | 100 | no |
| 229 | **21.3** | **678.8** | 10.0 | 200.2 | 900 | 884 | 150 | 700 | 20 | 100 | 15 | 260 | 220 | no |
| 230 | **22.1** | **231.2** | 9.8 | 200.9 | 900 | 884 | 130 | 750 | 20 | 100 | 15 | 260 | 220 | no |
| 231 | **22.2** | **433.8** | 9.8 | 200.0 | 900 | 884 | 130 | 750 | 20 | 100 | 30 | 260 | 220 | no |
| 232 | **23.2** | **50.3** | 10.0 | 200.0 | 900 | 884 | 130 | 750 | 20 | 100 | 3 | 260 | 220 | no |
| 304 | **19.24** | **258.42** | 9.84 | 199.7 | 900 | 884 | 130 | 700 | 20 | 100 | 15 | 260 | 220 | yes |
| 313 | **22.51** | **1001.62** | 10.45 | 199.1 | 900 | 884 | 130 | 700 | 1.9 | 500 | 15 | 210 | 100 | no |
| 314 | **21.33** | **673.7** | 9.85 | 200.2 | 900 | 884 | 130 | 700 | 1.7 | 500 | 5 | 210 | 100 | no |
| 315 | **19.85** | **291.87** | 9.89 | 200.1 | 800 | 787 | 130 | 650 | 1.7 | 500 | 5 | 210 | 100 | no |
| 316 | **17.84** | **194.07** | 10.27 | 200.0 | 750 | 738 | 130 | 600 | 1.8 | 500 | 5 | 210 | 100 | no |
| 317 | **22.09** | **247.77** | 9.75 | 199.9 | 775 | 762 | 130 | 625 | 1.8 | 500 | 5 | 210 | 100 | no |
| 318 | **18.23** | **148.99** | 9.88 | 200.3 | 800 | 787 | 130 | 650 | 1.8 | 500 | 2.5 | 210 | 100 | no |
| 319 | **8.38** | **896.8** | 4.99 | 200.3 | 800 | 787 | 150 | 550 | 1.8 | 500 | 5 | 210 | 100 | no |
| 320 | **10.13** | **3.12** | 10.18 | 199.6 | 800 | 787 | 130 | 650 | 950 | 500 | 5 | 210 | 100 | no |
| 323 | **20.41** | **998.41** | 9.98 | 200.5 | 900 | 884 | 130 | 700 | 1.9 | 500 | 15 | 210 | 100 | no |
| 328 | **18.62** | **301.95** | 9.70 | 199.6 | 900 | 884 | 130 | 700 | 1.8 | 500 | 0 | 210 | 100 | no |
| 329 | **20.07** | **262.58** | 9.85 | 199.6 | 900 | 831 | 130 | 700 | 1.8 | 500 | 0 | 210 | 100 | no |
| 330 | **18.73** | **267.06** | 9.74 | 200.6 | 900 | 864 | 130 | 700 | 1.8 | 500 | 0 | 210 | 100 | no |
| 331 | **3.3** | **277.58** | 2.40 | 50.0 | 900 | 884 | 130 | 700 | 1.8 | 500 | 0 | 210 | 100 | no |
| 332 | **20.09** | **261.78** | 10.49 | 199.0 | 900 | 856 | 130 | 700 | 1.8 | 500 | 0 | 210 | 100 | no |
| 333 | **20.07** | **264.6** | 10.18 | 199.4 | 900 | 851 | 130 | 700 | 1.8 | 500 | 0 | 210 | 100 | no |
| 334 | **16.51** | **1002.78** | 9.84 | 200.0 | 900 | 884 | 130 | 700 | 1.8 | 500 | 15 | 210 | 100 | no |
